# Maternal anaemia and the risk of postpartum haemorrhage: a cohort analysis of data from the WOMAN-2 trial

**DOI:** 10.1016/S2214-109X(23)00245-0

**Published:** 2023-06-27

**Authors:** The WOMAN-2 trial collaborators, Raoul Mansukhani, Haleema Shakur-Still, Rizwana Chaudhri, Folasade Bello, Projestine Muganyizi, Aasia Kayani, Kiran Javaid, Olujide Okunade, Oladapo Olayemi, Alice Kawala, Rose Temba, Alia Bashir, Amber Geer, Ansa Islam, Danielle Prowse, Eni Balogun, Francis Joseph, Haleema Yasmin, Mehnaz Khakwani, Mojisola Mobolaji-Ojibara, Najma Ghaffar, Olorunfemi Owa, Riffat Jaleel, Ruqqia Sultana, Saba Khan, Shahida Magsi, Shaista Abro, Shakila Yasmin, Shamila Munir, Shamsa Humayun, Shehla Noor, Sobia Luqman, Syeda Ali, Uzma Afridi, Vincent Tarimo, Ian Roberts

## Abstract

**Background:**

Worldwide, more than half a billion women of reproductive age are anaemic. Each year, about 70 000 women who give birth die from postpartum haemorrhage. Almost all deaths are in low-income or middle-income countries. We examined the association between anaemia and the risk of postpartum haemorrhage.

**Methods:**

We did a prospective cohort analysis of data from the World Maternal Antifibrinolytic-2 (WOMAN-2) trial. This trial enrols women with moderate or severe anaemia giving birth vaginally in hospitals in Pakistan, Nigeria, Tanzania, and Zambia. Moderate anaemia was defined as a haemoglobin concentration of 70–99 g/L and severe anaemia as less than 70 g/L. Hospitals in each country where anaemia in pregnancy is common were identified from a network established during previous obstetric trials. Women who were younger than 18 years without permission provided by a guardian, had a known tranexamic acid allergy, or developed postpartum haemorrhage before the umbilical cord was cut or clamped were excluded from the study. Prebirth haemoglobin, the exposure, was measured after hospital arrival and just before giving birth. Postpartum haemorrhage, the outcome, was defined in three ways: (1) clinical postpartum haemorrhage (estimated blood loss ≥500 mL or any blood loss sufficient to compromise haemodynamic stability); (2) WHO-defined postpartum haemorrhage (estimated blood loss of at least 500 mL); and (3) calculated postpartum haemorrhage (calculated estimated blood loss of ≥1000 mL). Calculated postpartum haemorrhage was estimated from the peripartum change in haemoglobin concentration and bodyweight. We used multivariable logistic regression to examine the association between haemoglobin and postpartum haemorrhage, adjusting for confounding factors.

**Findings:**

Of the 10 620 women recruited to the WOMAN-2 trial between Aug 24, 2019, and Nov 1, 2022, 10 561 (99·4%) had complete outcome data. 8751 (82·9%) of 10 561 women were recruited from hospitals in Pakistan, 837 (7·9%) from hospitals in Nigeria, 525 (5·0%) from hospitals in Tanzania, and 448 (4·2%) from hospitals in Zambia. The mean age was 27·1 years (SD 5·5) and mean prebirth haemoglobin was 80·7 g/L (11·8). Mean estimated blood loss was 301 mL (SD 183) for the 8791 (83·2%) women with moderate anaemia and 340 mL (288) for the 1770 (16·8%) women with severe anaemia. 742 (7·0%) women had clinical postpartum haemorrhage. The risk of clinical postpartum haemorrhage was 6·2% in women with moderate anaemia and 11·2% in women with severe anaemia. A 10 g/L reduction in prebirth haemoglobin increased the odds of clinical postpartum haemorrhage (adjusted odds ratio [aOR] 1·29 [95% CI 1·21–1·38]), WHO-defined postpartum haemorrhage (aOR 1·25 [1·16–1·36]), and calculated postpartum haemorrhage (aOR 1·23 [1·14–1·32]). 14 women died and 68 either died or had a near miss. Severe anaemia was associated with seven times higher odds of death or near miss (OR 7·25 [95% CI 4·45–11·80]) than was moderate anaemia.

**Interpretation:**

Anaemia is strongly associated with postpartum haemorrhage and the risk of death or near miss. Attention should be given to the prevention and treatment of anaemia in women of reproductive age.

**Funding:**

The WOMAN-2 trial is funded by Wellcome and the Bill & Melinda Gates Foundation.

## Introduction

Worldwide, about 500 million women of reproductive age are anaemic and 20 million are severely anaemic.^1^ The prevalence of anaemia is highest in western and central Africa and in south Asia, where about half of women of reproductive age are anaemic.^2^ Poor access to a healthy diet and untreated heavy menstrual bleeding, compounded by exposure to chronic infectious diseases (HIV, malaria, tuberculosis, or intestinal parasites), are believed to be the main causes.^1^ The consequences of anaemia include reduced work capacity in adults and poor cognitive and motor development in children.^3^ Severe anaemia in pregnancy increases the risk of maternal and infant death.^4^, ^5^

Postpartum haemorrhage, often defined as losing 500 mL or more of blood in the 24 h after giving birth,^6^ causes about 70 000 maternal deaths worldwide each year.^7^ Most deaths from postpartum haemorrhage are in low-income or middle-income countries.^7^ A systematic review and meta-analysis included all available evidence worldwide published before August, 2019, to investigate the association between prenatal anaemia and postpartum haemorrhage.^8^ The studies had a small sample size of low methodological quality, and considered anaemia as a categorical variable.^9^, ^10^, ^11^, ^12^, ^13^, ^14^ The systematic review concluded that severe anaemia increased the risk of postpartum haemorrhage, but found no statistical association between mild or moderate anaemia and the risk of postpartum haemorrhage. We examined the continuous association between prebirth haemoglobin and the risk of postpartum haemorrhage in a large cohort of women from Pakistan, Nigeria, Tanzania, and Zambia. The advantage of examining anaemia as a continuous variable is that demonstration of a monotonic biological gradient is more suggestive of a causal relationship. This analysis also allows determination of threshold effects.

**Research in context**

**Evidence before this study**

A systematic review and meta-analysis of the association between prenatal anaemia and postpartum haemorrhage was published in 2019. The review reported the results of a search of MEDLINE, Scopus, ClinicalTrials.gov, PROSPERO, Embase, and the Cochrane Central Register of Controlled Trials, with the search terms “anaemia”, “haemoglobin”, “postpartum haemorrhage”, and “postpartum bleeding”. The review found that severe anaemia was associated with an increased risk of postpartum haemorrhage (odds ratio [OR] 3·54 95% CI [1·20–10·4]) but there was no statistical evidence of an association with moderate (OR 2·09 [0·40–11·1]) or mild (OR 0·60 [0·31–1·17]) anaemia. The included studies were small, of low methodological quality, and used a variety of different postpartum haemorrhage definitions. Anaemia was examined as a categorical variable with no dose–response analysis.

**Added value of this study**

To our knowledge, this is the first study to examine the association between prebirth haemoglobin and postpartum haemorrhage, with haemoglobin as a continuous variable. This was possible because of the large sample size (n=10 561). The study participants were from low-income and middle-income countries where almost all deaths from postpartum haemorrhage occur. We controlled for confounding factors and there were negligible missing data. We found that with decreasing maternal haemoglobin concentration, the risk of postpartum haemorrhage increases monotonically. A 10 g/L decrease in prebirth haemoglobin is associated with increased odds for clinician-diagnosed postpartum haemorrhage. Women with severe anaemia had seven-fold increased odds of death or near miss compared with women with moderate anaemia. Our results were robust to different definitions of postpartum haemorrhage and to correction for haemoglobin measurement error.

**Implications of all the available evidence**

Anaemia is strongly associated with postpartum haemorrhage, with a dose–response relationship. Anaemia also increases the risk of death from postpartum haemorrhage and near miss. Attention should be given to preventing and treating anaemia in women of reproductive age.

## Methods

### Study design and participants

In our prospective cohort analysis**,** we used data from the World Maternal Antifibrinolytic-2 (WOMAN-2) trial, an ongoing randomised, placebo-controlled trial done in Pakistan, Nigeria, Tanzania, and Zambia.^15^ Participants were recruited from hospitals in each country where anaemia in pregnancy is common, and these were identified from a network established during previous obstetric trials. The WOMAN-2 trial includes women with moderate (haemoglobin concentration 70–99 g/L) or severe anaemia (haemoglobin concentration <70 g/L), who have given birth vaginally and for whom the responsible clinician is substantially uncertain whether to use tranexamic acid. Information about the woman's health and pregnancy status (including prebirth haemoglobin concentrations) were obtained as part of the assessment for trial entry. Women who were younger than 18 years without permission provided by a guardian, had a known tranexamic acid allergy, or developed postpartum haemorrhage before the umbilical cord was cut or clamped were excluded from the study. Women were weighed after admission and before giving birth. If scales were unavailable, we used the weight recorded at the most recent antenatal clinic visit. Clinicians were asked to measure postpartum haemoglobin within 24 h of the birth. The WOMAN-2 trial protocol^15^ was approved by the London School of Hygiene & Tropical Medicine's Ethics Committee (reference 15194). We obtained informed consent from women if their physical and mental capacity allowed. If a woman could not give consent, we obtained proxy consent from a relative or representative. If no proxy was available, then if local regulation allowed, we deferred or waived the consent. In these cases, we told the woman about the trial as soon as possible and obtained consent for use of the data collected. The consent procedures are described in detail in the WOMAN-2 trial protocol.^15^

### Procedures and outcomes

The exposure was prebirth haemoglobin concentration and the outcome was postpartum haemorrhage defined in one of three ways: (1) clinical postpartum haemorrhage, (2) WHO-defined postpartum haemorrhage, and (3) calculated postpartum haemorrhage. Firstly, clinical postpartum haemorrhage is diagnosed by a clinician usually because the patient has an estimated blood loss of at least 500 mL, or any blood loss sufficient to compromise haemodynamic stability within 24 h of the birth. Clinicians at each site were trained to estimate blood loss by the lead obstetrician and the trial team. We used pictorial guides, with photographs of soiled pads and bed sheets, to aid visual estimation of blood loss. Haemodynamic instability was based on signs such as low blood pressure, tachycardia, or reduced urine output. Secondly, we used the 1989 WHO working group definition for WHO-defined postpartum haemorrhage measurements, which is blood loss of at 500 mL or more from the genital tract within 24 h of a vaginal birth.^16^ The visual estimation was also based on the training. Finally, we used calculated postpartum haemorrhage measurements. Women with severe anaemia can appear unwell during and after birth regardless of the quantity of blood lost. Clinicians might overestimate blood loss in these women. We therefore used an objective measure of blood loss based on peripartum haemoglobin concentration.^17^, ^18^ We calculated estimated blood loss as the product of estimated blood volume and relative peripartum change in haemoglobin. Estimated blood volume (in litres) was obtained by multiplying weight in kg by 0·085. We corrected for the effect of transfusion on postpartum haemoglobin using the method described by Roubinian and colleagues.^19^ We classified a woman as having calculated postpartum haemorrhage if her calculated estimated blood loss was 1000 mL or more.

Because anaemia reduces the oxygen-carrying capacity of blood, women with anaemia cannot tolerate the same volume of bleeding as healthy women and become shocked after a smaller blood loss. Given the lack of an established definition of postpartum haemorrhage in women with anaemia, before conducting this study, we examined different definitions of postpartum haemorrhage in terms of their specificity for substantial bleeding (defined as shock index ≥1·0) and their association with fatigue, physical endurance, and breathlessness.^20^ We found that a clinical diagnosis of postpartum haemorrhage was highly specific for clinical signs of shock (95% specificity for shock index ≥1) and associated with worse maternal function. Estimated blood loss of 500 mL or more (WHO-defined) and calculated postpartum haemorrhage had similarly high specificity and were also associated with worse maternal functioning. We measured haemoglobin in capillary blood using a portable HemoCue 201+.

### Statistical analysis

We reported the cumulative incidence (risk) of postpartum haemorrhage over the observation period (24 h after birth). We then used multivariable logistic regression to examine the association between prebirth haemoglobin concentration and postpartum haemorrhage. The 10 g/L decrease in haemoglobin was obtained as an explanatory variable in the multivariable logistic regression model. To account for clustering, we included a random effect at the site level and a fixed effect for country. The same confounding factors were used in the models for each of the three definitions for postpartum haemorrhage. From the obstetric literature, we identified factors likely to be associated with maternal anaemia and the risk of postpartum haemorrhage. We described our causal assumptions using a directed acyclic graph (appendix p 1). We examined the association between the identified factors and postpartum haemorrhage using odds ratios (ORs) and 95% CIs. In our multivariable analyses, we controlled for BMI, previous postpartum haemorrhage, number of fetuses, parity, any antepartum haemorrhage, previous caesarean sections, placenta abnormalities, current infection, induction or augmentation of labour, pre-eclampsia, episiotomy, operative vaginal delivery, prolonged labour (labour lasting 20 h or more for first time mothers and 14 h or more for women who had previously given birth), and macrosomia (birthweight >4000 g). We estimated ORs and 95% CIs for the association between haemoglobin concentration and postpartum haemorrhage after controlling for these factors. We checked for collinearity using variance inflation factors, with a score of 4 or more considered to indicate collinearity. To examine whether antepartum haemorrhage or an abnormal placenta might modify the effect of haemoglobin concentration on clinical postpartum haemorrhage, we created a combined variable for both exposures. We added an interaction term between haemoglobin and this combined variable to our multivariable model. Finally, we calculated a p value for heterogeneity using a Wald test. We calculated an OR for the association between severe anaemia and maternal death or near miss. WHO defines a maternal near miss as ‘‘a woman who nearly died but survived a complication that occurred during pregnancy, childbirth or within 42 days of termination of pregnancy’’.^21^ The complications used for a near miss are shown in the appendix (p 2). We used a *t* test and Satterthwaite's approximation formula to test the hypothesis that estimated blood loss is independent of anaemia severity.

We examined the association between haemoglobin concentration and postpartum haemorrhage stratified by the presence or absence of other risk factors for postpartum haemorrhage. We created an indicator variable for having one or more risk factors. We used crude logistic regression to estimate the association between haemoglobin and clinical postpartum haemorrhage. We added polynomial terms for haemoglobin and used likelihood ratio tests to see if this improved the model fit. We used a threshold of p<0·05. We then added interaction terms for the indicator variable and all haemoglobin terms to our model. We checked if interaction terms improved fit in a likelihood ratio test with a threshold of p<0·05.

We also corrected for haemoglobin measurement error. The COMPARE study validated haemoglobin measurements obtained using capillary HemoCue samples against a laboratory gold standard in 5724 women who were aged 18 years or older and eligible to give blood through the UK's National Health Service donation service.^22^ We contacted the COMPARE study authors for details about their regression model for the difference versus the mean of haemoglobin measured by capillary HemoCue and the gold standard (appendix p 5). We used the coefficients and variance covariance matrix from their model to create a joint distribution. Regression coefficients were obtained by randomly sampling from this joint distribution. Gaussian error terms with mean of zero and variance equal to the mean squared error from COMPARE's regression model were also generated. These coefficients and error terms were used to impute gold standard haemoglobin measurements. We then randomly sampled our dataset with replacement. An OR for the effect of a 10 g/L reduction of haemoglobin on risk of postpartum haemorrhage was calculated using the imputed gold standard measurements. We repeated the aforementioned steps 10 000 times. Our central estimate and confidence intervals were the median, and the 2·5% and 97·5% centiles of our 10 000 samples. Further details of how we corrected for protentional HemoCue inaccuracy are provided in the appendix (p 4). For cases in which an analysis had a missing value for a relevant variable, participants were excluded. All data analysis was performed using R (version 4.3.0).

### Role of the funding source

The funders of the study had no role in study design, data collection, data analysis, data interpretation or writing of the report.

## Results

A total of 10 620 women were recruited to the WOMAN-2 trial between Aug 24, 2019, and Nov 1, 2022, of whom 10 561 (99·4%) had complete outcome data. 8751 (82·9%) of 10 561 women were recruited from hospitals in Pakistan, 837 (7·9%) from hospitals in Nigeria, 525 (5·0%) from hospitals in Tanzania, and 448 (4·2%) from hospitals in Zambia. Baseline characteristics are presented in table 1. Patient characteristics by country are shown in the appendix (pp 6–17). Data were missing for BMI (two women), estimated blood loss (six women), and postpartum haemoglobin (149 women). We estimated loss to follow-up as less than 0·1 % (appendix p 3). The mean age was 27·1 years (SD 5·5) and the mean bodyweight was 66·2 kg (9·3). Weight was measured for 5744 (54·4%) women and estimated on the basis of the most recent antenatal visit for 4817 (45·6%). Mean prebirth haemoglobin was 80·7 g/L (SD 11·8).

**Table 1:** Baseline characteristics

	**Participants (n=10 561)**
**Haemoglobin (g/L)**	
Mean (SD)	80·7 (11·8)
Moderately anaemic	8791 (83·2 %)
Severely anaemic	1770 (16·8%)
**Country**	
Pakistan	8751 (82·9%)
Nigeria	837 (7·9 %)
Zambia	448 (4·2%)
Tanzania	525 (5·0%)
**Age, years**	
Mean (SD)	27·1 (5·5)
<20	572 (5·4%)
20–29	6361 (60·2%)
30–39	3355 (31·8%)
≥40	273 (2·6%)
**BMI, kg/m^2^**	
Mean (SD)	26·4 (3·8)
<25	3933 (37·2%)
25–30	4924 (46·6%)
30–39	1665 (15·8%)
≥40	37 (0·4%)
Missing	2 (<0·1%)
**Weight, kg**	
Mean (SD)	66·2 (9·3)
<55	875 (8·3%)
55–64	3582 (33·9%)
65–74	4208 (39·8%)
≥75	1896 (18·0%)
**Number of fetuses**	
1	10 187 (96·5%)
2	363 (3·4%)
3	11 (0·1%)
**Parity (includes this pregnancy)**	
1	3490 (33·0%)
2–4	4984 (47·2%)
≥5	2087 (19·8%)
**Previous postpartum haemorrhage**	
Yes	113 (1·1%)
No	6808 (64·5%)
No previous birth	3486 (33·0%)
Missing	154 (1·5%)
**Placenta abnormalities**	
Abruption	332 (3·1%)
Previa	31 (0·3%)
Abruption, previa	5 (<0·1%)
Accreta	1 (<0·1%)
None	10 192 (96·5%)
**Any antepartum haemorrhage**	
Yes	337 (3·2%)
No	10 224 (96·8%)
**Number of previous caesarean sections**	
0	10 023 (94·9%)
1	512 (4·8%)
2	20 (0·2%)
3	6 (0·1%)
**Current infection status**	
HIV	173 (1·6%)
Hepatitis	118 (1·1%)
Malaria	16 (0·2%)
Syphilis	11 (0·1%)
Other	72 (0·7%)
None	10 185 (96·4%)
**Hypertensive disease**	
Eclampsia	27 (0·3%)
Pre-eclampsia	204 (1·9%)
Pre-existing hypertension	45 (0·4%)
Pregnancy-induced hypertension	540 (5·1%)
None	9749 (92·3%)
**Assisted delivery**	
Forceps	96 (0·9%)
Ventouse	160 (1·5%)
Other	53 (0·5%)
None	10 256 (97·1%)
**Induction**	
Artificial membrane rupture	328 (3·1%)
Mechanical method	380 (3·6%)
Membrane sweep	155 (1·5%)
Oxytocin	45 (0·4%)
Misoprostol	341 (3·2%)
Prostaglandin	386 (3·7%)
**None**	**9167 (86·8%)**
**Augmentation**	
Oxytocin	3162 (29·9%)
Other	259 (2·5%)
None	7201 (68·2%)
**Episiotomy**	
Yes	3370 (31·9%)
No	7191 (68·1%)
**Long labour**	
Yes	727 (6·9%)
No	9834 (93·1%)
**Known macrosomia**	
Yes	162 (1·5%)
No	10 399 (98·5%)

Data are mean (SD) or n (%). For infection status, hypertensive disease, assisted delivery, induction, and augmentation, proportions will not sum to 100% as responses are not mutually exclusive. For other variables proportions might not sum to 100% due to rounding.

742 (7·0%) women had a clinical postpartum haemorrhage. Mean prebirth haemoglobin for women with clinical postpartum haemorrhage was 76·4 g/L (SD 13·8). The risk of clinical postpartum haemorrhage was 6·2% (544 of 8791) in women with moderate anaemia and 11·2% (198 of 1770) in women with severe anaemia. The primary diagnosis for postpartum haemorrhage by anaemia status is shown in table 2. The most common diagnoses were uterine atony (361 [66%] of 544 participants with moderate anaemia and 134 [68%] of 198 with severe anaemia), tears (101 [19%] of 544 with moderate anaemia and 23 [12%] of 198 with severe anaemia), and retained placenta tissue (58 [11%] of 544 with moderate anaemia and 20 [10%] of 198 with severe anaemia).

**Table 2:** Primary cause of postpartum haemorrhage

	**Moderate anaemia (n=544)**	**Severe anaemia (n=198)**
Atony	361 (66%)	134 (68%)
Tears	101 (19%)	23 (12%)
Retained placenta tissue	58 (11%)	20 (10%)
Placenta implantation abnormalities	3 (1%)	7 (4%)
Uterine rupture	1 (<1%)	0
Other	8 (1%)	9 (5%)
Unknown	12 (2%)	5 (3%)

Data are n (%).

14 women died (six with moderate anaemia and eight with severe anaemia), in whom the mean prebirth haemoglobin was 68·4 g/L (SD 19·1). Primary causes of death were bleeding (eight women), anaemic cardiac failure (two women), acute respiratory distress syndrome (two women), HELLP syndrome (one woman; defined as pre-eclampsia characterised by microangiopathic haemolytic anaemia, elevated liver enzymes, and low platelets), and sepsis (one woman). Compared with moderate anaemia, severe anaemia was associated with an increased odds of death (OR 6·65 [95% CI 2·30–19·18]). For the nine women who died and had clinical postpartum haemorrhage, mean haemoglobin was 70·0 g/L (SD 17·4). For the eight women who died and had bleeding as their cause of death, mean haemoglobin was 66·6 g/L (SD 17·3). 68 women died or had a near miss, in whom mean haemoglobin was 65·0 g/L (SD 18·1). Severe anaemia was associated with a seven times higher increased odds of death or near miss (OR 7·25 [95% CI 4·45–11·80]) than was moderate anaemia.

**Figure 1:**
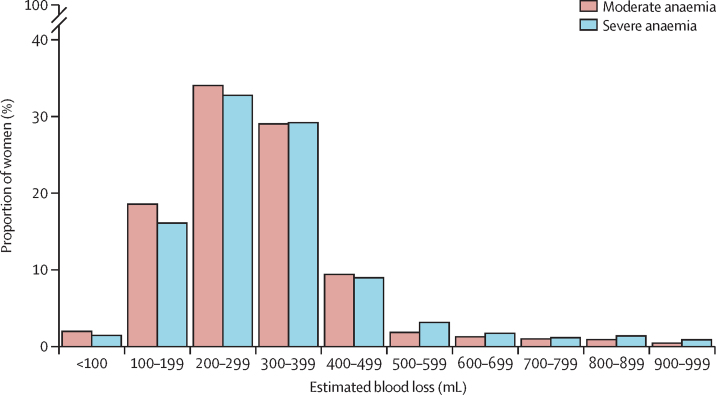
Bar chart of estimated blood loss stratified by anaemia status 170 women with estimated blood loss of 1 L or more and six women with a missing estimated blood loss value are omitted (n=10 385).

Estimated blood loss by anaemia severity is shown in figure 1. Mean estimated blood loss was larger (p<0·0001) in women with severe anaemia (340 mL [SD 288]) than in women with moderate anaemia (301 mL [183]). 22 (0·3%) of 8787 women with moderate anaemia and 22 (1·2%) of 1768 women with severe anaemia lost at least 1500 mL of blood.

Univariable and multivariable analyses of risk factors for postpartum haemorrhage are shown in Table 3, Table 4, Table 5. Univariable analyses show that a 10 g/L reduction in prebirth haemoglobin increases the odds of clinical postpartum haemorrhage (OR 1·36 [95% CI 1·27–1·46]), WHO-defined postpartum haemorrhage (OR 1·31 [1·22–1·41]), and calculated postpartum haemorrhage (OR 1·25 [1·16–1·34]). After controlling for potential confounding factors in our multivariable model, the association between prebirth haemoglobin and postpartum haemorrhage was slightly reduced. A 10 g/L reduction in prebirth haemoglobin increased the odds for clinical postpartum haemorrhage (with an adjusted [a] OR 1·29 [95% CI 1·21–1·38]), WHO-defined postpartum haemorrhage (aOR 1·25 [1·16–1·36]), and calculated postpartum haemorrhage (aOR 1·23 [1·14–1·32]).

**Table 3:** Univariable and multivariable analyses of risk factors for clinical postpartum haemorrhage in women with moderate or severe anaemia

		**Number of women with postpartum haemorrhage**	**OR (95% CI)**	**aOR (95% CI)**
10 g/L reduction in prebirth haemoglobin		742/10 561 (7·0%)	1·36 (1·27–1·46)	1·29 (1·21–1·38)
Country				
	Pakistan	601/8751 (6·9%)	1 (ref)	1 (ref)
	Nigeria	78/837 (9·3%)	1·47 (0·99–2·19)	2·17 (1·33–3·52)
	Tanzania	32/525 (6·1%)	0·95 (0·62–1·46)	1·43 (0·84–2·44)
	Zambia	31/448 (6·9%)	1·11 (0·90–1·38)	1·37 (0·91–2·05)
BMI, kg/m^2^				
	<25	284/3933 (7·2%)	1 (ref)	1 (ref)
	25–30	337/4924 (6·8%)	0·99 (0·84–1·18)	1·01 (0·83–1·23)
	30–39	117/1665 (7·0%)	1·04 (0·78–1·38)	0·97 (0·72–1·32)
	≥40	4/37 (10·8%)	1·60 (0·51–5·04)	1·51 (0·51–4·49)
Number of fetuses				
	1	672/10 187 (6·6%)	1 (ref)	1 (ref)
	≥2	70/374 (18·7%)	3·36 (2·58–4·38)	3·79 (2·86–5·01)
Parity (includes this pregnancy)				
	1	251/3490 (7·2%)	1 (ref)	1 (ref)
	2–4	310/4984 (6·2%)	0·84 (0·71–1·00)	1·06 (0·87–1·30)
	≥5	181/2087 (8·7%)	1·15 (0·92–1·44)	1·31 (1·01–1·70)
Previous postpartum haemorrhage				
	No or no previous birth	707/10 294 (6·9%)	1 (ref)	1 (ref)
	Yes	17/113 (15·0%)	2·34 (1·27–4·32)	2·16 (1·19–3·94)
Placenta abnormalities				
	No	632/10 192 (6·2%)	1 (ref)	1 (ref)
	Yes	110/369 (29·8%)	6·85 (5·13–9·16)	3·63 (2·20–6·01)
Any antepartum haemorrhage				
	No	651/10 224 (6·4%)	1 (ref)	1 (ref)
	Yes	91/337 (27·0%)	5·84 (4·34–7·87)	1·52 (0·93–2·47)
Previous caesarean sections				
	No	708/10 023 (7·1%)	1 (ref)	1 (ref)
	Yes	34/538 (6·3%)	0·88 (0·58–1·33)	0·82 (0·54–1·24)
Current infection				
	No	704/10 185 (6·9%)	1 (ref)	1 (ref)
	Yes	38/376 (10·1%)	1·56 (0·81–2·99)	1·60 (0·92–2·77)
Hypertensive disease				
	No	610/9749 (6·3%)	1 (ref)	1 (ref)
	Yes	132/812 (16·3%)	3·01 (2·32–3·89)	2·12 (1·62–2·78)
Assisted delivery				
	No	688/10 256 (6·7%)	1 (ref)	1 (ref)
	Yes	54/305 (17·7%)	3·10 (2·25–4·27)	2·30 (1·58–3·34)
Augmentation or induction				
	No	413/6612 (6·2%)	1 (ref)	1 (ref)
	Yes	329/3949 (8·3%)	1·41 (1·15–1·74)	1·30 (1·04–1·64)
Episiotomy				
	No	480/7191 (6·7%)	1 (ref)	1 (ref)
	Yes	262/3370 (7·8%)	1·25 (1·03–1·51)	1·67 (1·32–2·12)
Long labour				
	No	682/9834 (6·9%)	1 (ref)	1 (ref)
	Yes	60/727 (8·3%)	1·13 (0·91–1·42)	1·13 (0·90–1·42)
Known macrosomia				
	No	719/10 399 (6·9%)	1 (ref)	1 (ref)
	Yes	23/162 (14·2%)	2·21 (1·47–3·32)	2·19 (1·44–3·32)

The multivariable model controls for haemoglobin, country, BMI, number of fetuses, parity, previous postpartum haemorrhage, placenta abnormalities, any antepartum haemorrhage, previous caesarean sections, any current infection, hypertensive disease, assisted delivery, augmentation or induction, episiotomy, long labour and known macrosomia. n=10 561 for the univariable model and n=10 405 for the multivariable model. aOR=adjusted odds ratio.

**Table 4:** Univariable and multivariable analyses of risk factors for WHO-defined postpartum haemorrhage in women with moderate or severe anaemia

		**Number of women with postpartum haemorrhage**	**OR (95% CI)**	**aOR (95% CI)**
10 g/L reduction in prebirth haemoglobin		810/10 555 (7·7%)	1·31 (1·22–1·41)	1·25 (1·16–1·36)
Country				
	Pakistan	633/8747 (7·2%)	1 (ref)	1 (ref)
	Nigeria	97/835 (11·6%)	1·77 (1·30–2·41)	2·30 (1·59–3·32)
	Tanzania	44/525 (8·4%)	1·40 (0·76–2·58)	1·91 (1·14–3·22)
	Zambia	36/448 (8·0%)	1·22 (1·02–1·46)	0·88 (0·64–1·22)
BMI, kg/m^2^				
	<25	297/3929 (7·6%)	1 (ref)	1 (ref)
	25–30	369/4922 (7·5%)	1·00 (0·86–1·17)	1·02 (0·85–1·21)
	30–39	140/1665 (8·4%)	1·16 (0·84–1·61)	1·12 (0·77–1·63)
	≥40	4/37 (10·8%)	1·37 (0·43–4·38)	1·21 (0·38–3·82)
Number of fetuses				
	1	733/10 181 (7·2%)	1 (ref)	1 (ref)
	≥2	77/374 (20·6%)	3·33 (2·61–4·26)	3·74 (2·89–4·84)
Parity (includes this pregnancy)				
	1	285/3488 (8·2%)	1 (ref)	1 (ref)
	2–4	338/4982 (6·8%)	0·80 (0·70–0·92)	1·04 (0·86–1·24)
	≥5	187/2085 (9·0%)	1·07 (0·86–1·33)	1·27 (0·97–1·65)
Previous postpartum haemorrhage				
	No or no previous birth	771/10 288 (7·5%)	1 (ref)	1 (ref)
	Yes	20/113 (17·7%)	2·51 (1·44–4·38)	2·38 (1·42–4·00)
Placenta abnormalities				
	No	698/10 186 (6·9%)	1 (ref)	1 (ref)
	Yes	112/369 (30·4%)	6·23 (4·84–8·02)	3·70 (2·20–6·22)
Any antepartum haemorrhage				
	No	717/10 218 (7·0%)	1 (ref)	1 (ref)
	Yes	93/337 (27·6%)	5·25 (3·90–7·07)	1·38 (0·80–2·36)
Previous caesarean sections				
	No	776/10 018 (7·7%)	1 (ref)	1 (ref)
	Yes	34/537 (6·3%)	0·79 (0·55–1·15)	0·74 (0·51–1·07)
Current infection				
	No	771/10 179 (7·6%)	1 (ref)	1 (ref)
	Yes	39/376 (10·4%)	1·37 (0·65–2·89)	1·38 (0·70–2·74)
Hypertensive disease				
	No	667/9743 (6·8%)	1 (ref)	1 (ref)
	Yes	143/812 (17·6%)	2·91 (2·32–3·67)	2·11 (1·62–2·74)
Assisted delivery				
	No	758/10 250 (7·4%)	1 (ref)	1 (ref)
	Yes	52/305 (17·0%)	2·63 (1·87–3·70)	1·85 (1·27–2·69)
Augmentation or induction				
	No	459/6607 (6·9%)	1 (ref)	1 (ref)
	Yes	351/3948 (8·9%)	1·39 (1·17–1·66)	1·28 (1·05–1·55)
Episiotomy				
	No	510/7187 (7·1%)	1 (ref)	1 (ref)
	Yes	300/3368 (8·9%)	1·29 (1·10–1·52)	1·73 (1·42–2·12)
Long labour				
	No	745/9829 (7·6%)	1 (ref)	1 (ref)
	Yes	65/726 (9·0%)	1·14 (0·93–1·39)	1·11 (0·90–1·37)
Known macrosomia				
	No	788/10 393 (7·6%)	1 (ref)	1 (ref)
	Yes	22/162 (13·6%)	1·88 (1·22–2·89)	1·81 (1·16–2·83)

WHO-defined postpartum haemorrhage is defined as blood loss of at least 500 mL in the first 24 h after giving birth. The multivariable model controls for haemoglobin, country, BMI, number of fetuses, parity, previous postpartum haemorrhage, placenta abnormalities, any antepartum haemorrhage, previous caesarean sections, any current infection, hypertensive disease, assisted delivery, augmentation or induction, episiotomy, long labour, and known macrosomia. n=10 555 for the univariable model and n=10 399 for the multivariable model. aOR=adjusted odds ratio.

**Table 5:** Univariable and multivariable analyses of risk factors for calculated postpartum haemorrhage in women with moderate or severe anaemia

		**Number of women with postpartum haemorrhage**	**OR (95% CI)**	**aOR (95% CI)**
10 g/L reduction in prebirth haemoglobin		926/10408 (8·9%)	1·25 (1·16–1·34)	1·23 (1·14–1·32)
Country				
	Pakistan	664/8624 (7·7%)	1 (ref)	1 (ref)
	Nigeria	90/820 (11·0%)	1·48 (1·03–2·11)	1·85 (1·32–2·58)
	Tanzania	90/523 (17·2%)	2·46 (1·65–3·66)	2·97 (2·18–4·06)
	Zambia	82/441 (18·6%)	2·80 (2·27–3·47)	2·94 (2·33–3·71)
BMI, kg/m^2^				
	<25	304/3853 (7·9%)	1 (ref)	1 (ref)
	25–30	444/4858 (9·1%)	1·27 (1·05–1·54)	1·27 (1·08–1·50)
	30–39	173/1659 (10·4%)	1·45 (1·19–1·76)	1·40 (1·14–1·72)
	≥40	4/36 (11·1%)	1·09 (0·34–3·47)	1·02 (0·39–2·69)
Number of fetuses				
	1	862/10 040 (8·6%)	1 (ref)	1 (ref)
	≥2	64/368 (17·4%)	1·99 (1·55–2·54)	1·98 (1·54–2·53)
Parity (includes this pregnancy)				
	1	341/3441 (9·9%)	1 (ref)	1 (ref)
	2–4	402/4905 (8·2%)	0·84 (0·72–0·99)	0·85 (0·68–1·07)
	≥5	183/2062 (8·9%)	0·99 (0·84–1·17)	0·91 (0·70–1·18)
Previous postpartum haemorrhage				
	No or no previous birth	887/10 147 (8·7%)	1 (ref)	1 (ref)
	Yes	20/109 (18·3%)	2·19 (1·41–3·39)	2·10 (1·38–3·20)
Placenta abnormalities				
	No	829/10 047 (8·3%)	1 (ref)	1 (ref)
	Yes	97/361 (26·9%)	4·04 (3·06–5·34)	2·02 (1·22–3·35)
Any antepartum haemorrhage				
	No	835/10 076 (8·3%)	1 (ref)	1 (ref)
	Yes	91/332 (27·4)	4·13 (3·08–5·53)	1·90 (1·14–3·19)
Previous caesarean sections				
	No	872/9882 (8·8%)	1 (ref)	1 (ref)
	Yes	54/526 (10·3%)	1·20 (0·85–1·69)	1·16 (0·80–1·66)
Current infection				
	No	870/10 040 (8·7%)	1 (ref)	1 (ref)
	Yes	56/368 (15·2)	1·36 (1·00–1·85)	1·29 (0·98–1·69)
Hypertensive disease				
	No	790/9603 (8·2%)	1 (ref)	1 (ref)
	Yes	136/805 (16·9%)	2·21 (1·83–2·66)	1·67 (1·38–2·03)
Assisted delivery				
	No	859/10 107 (8·5%)	1 (ref)	1 (ref)
	Yes	67/301 (22·3%)	2·78 (1·98–3·89)	2·11 (1·56–2·85)
Augmentation or induction				
	No	551/6499 (8·5%)	1 (ref)	1 (ref)
	Yes	375/3909 (9·6%)	1·19 (1·00–1·41)	1·12 (0·93–1·35)
Episiotomy				
	No	625/7109 (8·8%)	1 (ref)	1 (ref)
	Yes	301/3299 (9·1%)	1·14 (0·97–1·34)	1·19 (0·95–1·48)
Long labour				
	No	863/9688 (8·9%)	1 (ref)	1 (ref)
	Yes	63/720 (8·8%)	0·89 (0·69–1·13)	0·84 (0·64–1·11)
Known macrosomia				
	No	896/10 248 (8·7%)	1 (ref)	1 (ref)
	Yes	30/160 (18·8%)	2·26 (1·52–3·36)	2·30 (1·56–3·41)

Calculated postpartum haemorrhage is defined as calculated estimated blood loss of at least 1000 mL. The multivariable model controls for haemoglobin, country, BMI, number of fetuses, parity, previous postpartum haemorrhage, placenta abnormalities, any antepartum haemorrhage, previous caesarean sections, any current infection, hypertensive disease, assisted delivery, augmentation or induction, episiotomy, long labour, and known macrosomia. n=10 408 for the univariable model and n=10 254 for the multivariable model. aOR=adjusted odds ratio.

Antepartum haemorrhage, placenta abnormalities, and current infection might be on the causal pathway between anaemia and postpartum haemorrhage in which case controlling for these factors would be inappropriate. When we conducted a post-hoc sensitivity analysis excluding these factors from the multivariable model (appendix p 18), the results were similar (clinical postpartum haemorrhage, aOR 1·40 [95% CI 1·30–1·50]). There was no evidence that antepartum haemorrhage or abnormal placenta modified the association between haemoglobin and clinical postpartum haemorrhage (p=0·31). A 10 g/L reduction in haemoglobin increased the odds of postpartum haemorrhage in 442 women with antepartum haemorrhage or abnormal placenta (aOR 1·41 [95% CI 1·19–1·68]) and in 10 119 women without these risk factors (aOR 1·26 [1·17–1·36]).

We found no evidence of variable collinearity. The maximum variance inflation factor was 3·0 for abnormal placenta in the multivariable model with the outcome clinical postpartum haemorrhage. When we corrected for HemoCue inaccuracy, a 10 g/L reduction in prebirth haemoglobin increased both the unadjusted (OR 1·30 [95% CI 1·22–1·40]) and the adjusted (aOR 1·22 [1·14–1·32]) odds of clinical postpartum haemorrhage.

**Figure 2:**
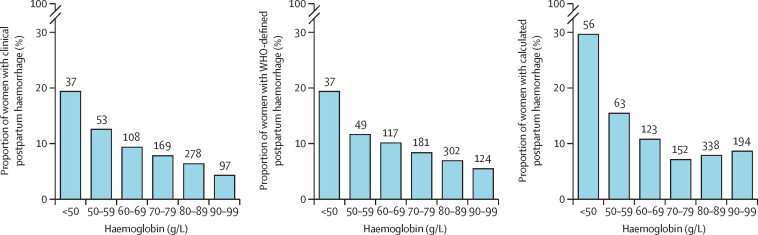
Percentage of women with postpartum haemorrhage versus prebirth haemoglobin concentration Clinical postpartum haemorrhage is defined as an estimated blood loss of at least 500 mL or any blood loss sufficient to compromise haemodynamic stability. WHO-defined postpartum haemorrhage is defined as blood loss of at least 500 mL in the first 24 h after giving birth. Calculated clinical postpartum haemorrhage is defined as calculated estimated blood loss of at least 1000 mL. For clinical postpartum haemorrhage, n=10 561; for WHO-defined postpartum haemorrhage, n=10 555; for calculated postpartum haemorrhage, n=10 408. The numbers of women with postpartum haemorrhage are displayed on the histogram bars.

The relationship between prebirth haemoglobin and postpartum haemorrhage is presented in figure 2. For all three postpartum haemorrhage definitions, as haemoglobin decreases, the risk of postpartum haemorrhage increases.

**Figure 3:**
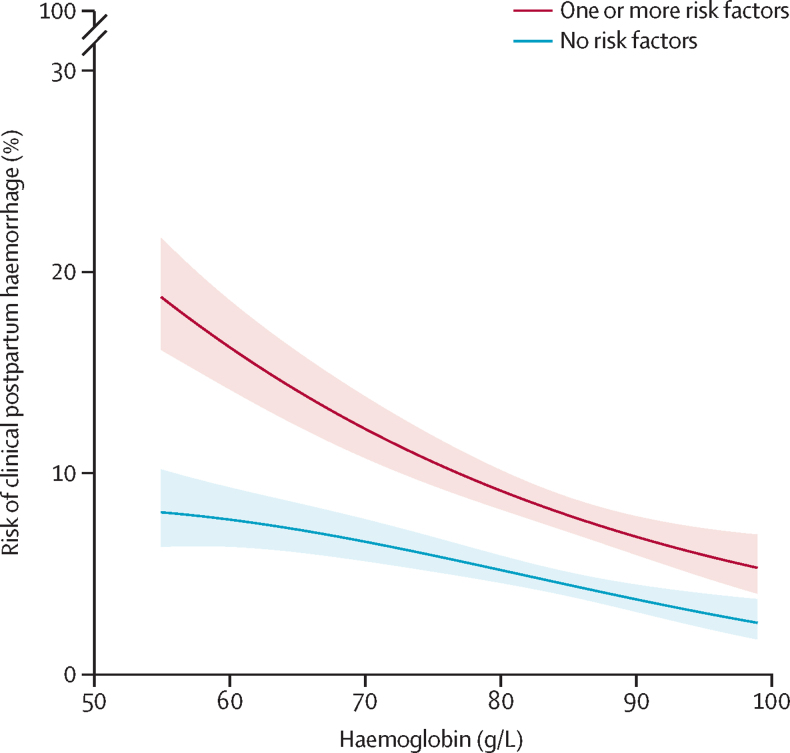
Relationship between haemoglobin and risk of clinical postpartum haemorrhage The risk of postpartum haemorrhage is calculated using logistic regression with a quadratic term for haemoglobin. The red is the postpartum haemorrhage risk in 4564 women with one or more risk factors (previous postpartum haemorrhage, abnormal placenta, antepartum haemorrhage, infection, multiple pregnancy, and primigravida). The blue line is the risk of postpartum haemorrhage in 5997 women without risk factors. The shaded areas are 95% CIs.

The association between prebirth haemoglobin and clinical postpartum haemorrhage in women with (n=4564) and without (n=5997) one or more risk factors is presented in figure 3. Our model includes a quadratic term for haemoglobin. For those with additional risk factors, a decrease in haemoglobin from 90 g/L to 70 g/L increased the risk of postpartum haemorrhage from 6·9% to 12·2% (a 77% increased risk). In those without risk factors, a decrease in haemoglobin from 90 g/L to 70 g/L increased the risk of postpartum haemorrhage from 3·8% to 6·6% (a 75% increased risk). Although the proportional effect of haemoglobin on risk of postpartum haemorrhage is similar in women with and without additional postpartum haemorrhage risk factors, there was some evidence of statistical heterogeneity (p=0·072).

## Discussion

A low prebirth haemoglobin concentration greatly increases the probability of postpartum haemorrhage. The association between prebirth haemoglobin and postpartum haemorrhage is similar regardless of how postpartum haemorrhage is defined. Women with severe anaemia also have a greatly increased risk of death or near miss.

To our knowledge, this is the largest cohort study of the association between prebirth haemoglobin and postpartum haemorrhage. The large sample size allowed us to examine maternal anaemia as a continuous rather than a categorical variable. Loss to follow-up was negligible and there were few missing data for any variable. Prebirth haemoglobin concentration was measured using capillary haemoglobinometry (HemoCue), which has been shown to be accurate and with the smallest bias in comparison with the reference standard. Postpartum haemorrhage was assessed clinically and objectively on the basis of measured haemoglobin change. The study dataset allowed us to control for many potential confounding factors for anaemia and postpartum haemorrhage risk.

Several cohort studies have shown that maternal anaemia increases the risk of postpartum haemorrhage and death. Daru and colleagues^4^ analysed data from the WHO Multi-country Survey and found that the odds of postpartum haemorrhage and death are higher for women with severe anaemia than those without severe anaemia. Shi and colleagues^5^ examined data from China's Hospital Quality Monitoring System and found that compared with no anaemia, all anaemia categories (mild, moderate, or severe) were associated with an increased risk of postpartum haemorrhage. A cohort study in Assam, India, investigated the association between anaemia and maternal outcomes in 1007 women giving birth in hospital and found that the odds of postpartum haemorrhage in women with severe anaemia were nine times higher than in women with mild or no anaemia.^9^ A cohort study in Norway showed that a prebirth haemoglobin concentration of less than 90 g/L was associated with a doubling of the odds for severe obstetric haemorrhage.^12^ Smaller cohort studies done in Senegal, Mali, and Tanzania also found an increased risk.^10^, ^23^ Our results confirm and extend the conclusions of these studies. However, unlike previous cohort studies in which anaemia was recorded as a binary or categorical variable, our study recorded true haemoglobin concentrations in all women, allowing us to assess the dose–response relation between prebirth haemoglobin and the risk of postpartum haemorrhage. Another major strength is that our study has negligible missing data on confounding variables and almost complete follow-up. The monotonic biological gradient between prebirth haemoglobin and postpartum haemorrhage risk observed in our study is suggestive of a causal relationship.

Although studies comparing haemoglobin concentration measured using capillary HemoCue and a reference standard laboratory measurement have shown that accuracy is high, some measurement error is inevitable.^22^ Using data from the COMPARE study^22^ to correct for HemoCue inaccuracy slightly attenuated our results. There will also be some inaccuracy in postpartum blood loss estimation. This is likely to attenuate the association between haemoglobin and postpartum haemorrhage risk. Although we adjusted for many potential confounding factors, there might be some confounding factors that we were unable to control for. Because all women in our study had moderate or severe anaemia, we could not investigate the association between haemoglobin and postpartum haemorrhage in women with mild anaemia. Although women were enrolled from hospitals in Pakistan, Nigeria, Zambia, and Tanzania, most were recruited in Pakistan. However, we see no biological reason why the association between anaemia and postpartum haemorrhage that we observed would not be widely generalisable to women giving birth out of hospital or in other countries. Our cohort study is part of an ongoing randomised controlled trial of the effect of tranexamic acid on the risk of postpartum haemorrhage. Tranexamic acid should not confound the association between prebirth haemoglobin and postpartum haemorrhage risk, but the possibility of a biological interaction between haemoglobin and tranexamic acid is open to question and will be examined in the WOMAN-2 trial.

There are several mechanisms through which anaemia could worsen postpartum bleeding. First, the increased heart rate and cardiac output caused by anaemia could increase blood flow from bleeding vessels.^24^ As haemoglobin falls, hypoxia-sensing cells in the aortic arch activate the sympathetic nervous system, increasing heart rate and stroke volume to maintain oxygen delivery. Second, the reduced blood viscosity from anaemia could result in increased blood flow. Whittaker and Winton showed that apparent blood viscosity decreases with decreasing haematocrit and Poiseuille's law predicts an increase in blood flow as viscosity falls.^25^ Third, anaemic blood clots might be more susceptible to fibrinolysis.^26^ Red blood cells appear to have an antifibrinolytic effect due to suppressed tPA-induced fibrinolysis in red blood cell-modified fibrin structures. Finally, it has been suggested that anaemia might cause uterine atony from impaired uterine oxygenation.^9^ However, isovolaemic reduction of haemoglobin to 50 g/L in healthy volunteers has no measurable effect on tissue oxygenation or blood lactate and uterine hypoxia appears to increase rather than decrease the strength of myometrial contraction.^24^, ^27^

Although the OR for a 10 g/L reduction in prebirth haemoglobin is smaller than for some other known risk factors for postpartum haemorrhage, because anaemia is a strong risk factor that is also highly prevalent worldwide, a substantial proportion of postpartum haemorrhage cases are likely to be due to anaemia. Nonetheless, anaemia was not recorded as the primary cause of postpartum haemorrhage in any of the 10 561 women in our study. Our results endorse the recommendation that every effort should be made to correct anaemia before delivery.^28^

## Data sharing

Following publication of the WOMAN-2 trial results, individual deidentified patient data, including a data dictionary, will be made available via our data sharing portal, The Free Bank of Injury and Emergency Research Data (freebird.lshtm.ac.uk) website, indefinitely.

Correspondence to: The WOMAN-2 trial collaborators, Global Health Trials Group, London School of Hygiene & Tropical Medicine, London WC1E 7HT, UK

**This online publication has been corrected. The corrected version first appeared at thelancet.com/lancetgh on July 18, 2023**

## Declaration of interests

We declare no competing interests.
