# Correction to *Lancet Glob Health* 2023; 11: e1249–59

**DOI:** 10.1016/S2214-109X(23)00321-2

**Published:** 2023-07-18

*The WOMAN-2 trial collaborators. Maternal anaemia and the risk of postpartum haemorrhage: a cohort analysis of data from the WOMAN-2 trial.* Lancet Glob Health *2023; **11:** e1249–59*—In this Article, the definition of moderate and severe anaemia has been updated in the Summary and the Methods section, and in the Methods section the value of 0·85 has been changed to 0·085. These corrections have been made as of July 18, 2023.

