# Supplementary material for: Maternal anaemia and the risk of postpartum haemorrhage: a cohort analysis of data from the WOMAN-2 trial
Source: Lancet Glob Health. 2023 Jun 27;11(8):e1249–59. doi: 10.1016/S2214-109X(23)00245-0 (PMC10353972; doi:10.1016/S2214-109X(23)00245-0)
Supplement: Supplementary appendix [file mmc1.pdf]

Web appendix

Web appendix figure 1: DAG indicating the potential causal relationship between haemoglobin concentration, postpartum haemorrhage and potential confounding factors. Abbreviations: Antepartum haemorrhage (APH), Body mass index (BMI)

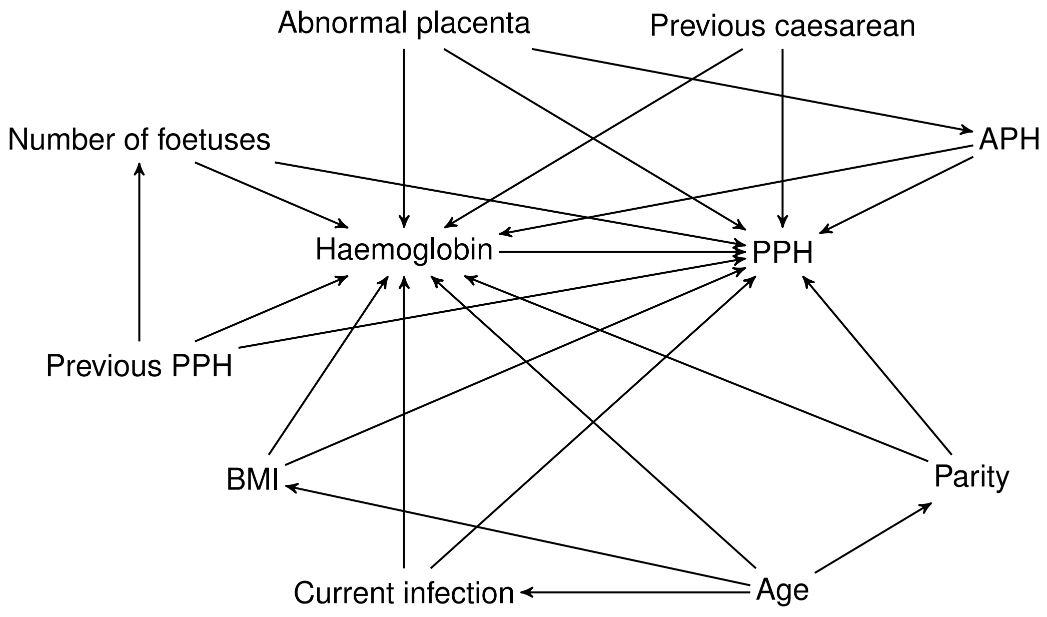

Web appendix section 1: World Health Organisation criteria for a near miss

A WHO maternal near miss occurs if a woman survives after being diagnosed with a condition from the criteria below during pregnancy, childbirth or within 42 days of termination of pregnancy[1].

Clinical criteria
Acute cyanosis
Gasping
Respiratory rate >40 or <6/min
Shock
Oliguria non-responsive to fluids or diuretics
Clotting failure
Loss of consciousness lasting ≥12 hours
Loss of consciousness AND absence of pulse/heartbeat
Stroke
Uncontrollable fit/total paralysis
Jaundice in the presence of pre-eclampsia

Laboratory-based criteria
Oxygen saturation <90% for ≥60 minutes
PaO2/FiO2 <200 mmHg
Creatinine ≥300 mmol/l or ≥3.5 mg/dl
Bilirubin>10 0 mmol/l or > 6.0 mg/dl
pH <7.1
Lactate >5
Acute thrombocytopenia (<50000 platelets)
Loss of consciousness AND the presence of glucose
ketoacids in urine

Management-based criteria
Use of continuous vasoactive drugs
Hysterectomy following infection or haemorrhage
Transfusion of ≥5 units red cell transfusion
Intubation and ventilation for ≥60 minutes not related to anaesthesia
Dialysis for acute renal failure
Cardio-pulmonary resuscitation (CPR)

In our data shock is defined as persistent systolic blood pressure <80 mmHg or a persistent systolic blood pressure <90 mmHg with a pulse rate of at least 120 bpm. Oliguria and clotting failure are assessed by a clinician. Stroke is diagnosed if the patient has a new focal neurological deficit with signs and symptoms lasting more than 24 hours. The vasoactive drugs administered were dopamine, epinephrine and norepinephrine. Pre-eclampsia is raised blood pressure and proteinuria. Gasping is a respiratory pattern where the breath is convulsively and audibly caught.

Web appendix section 2: Loss to follow-up

The WOMAN-2 trial is ongoing so loss to follow-up needs to be estimated.

To estimate loss to follow-up we divided the number of participants with a missing outcome record by the total number of patients with a completed baseline record. We only considered patients enrolled before 22nd September 2022. It is likely that sites will complete missing outcome forms for patients enrolled after that date.

% loss to follow up

= 100 * (patients with a missing outcome record)/ (patients with a baseline record)
(We only consider patients enrolled before 22nd September 2022)
=100* 10/ 10,057 = <0.1 %

Web appendix section 3: Correcting for haemoglobin mismeasurement

Background
Our study examines the association between prebirth haemoglobin and risk of postpartum haemorrhage (PPH) using WOMAN-2 trial data. Prebirth haemoglobin is measured using a capillary HemoCue 201+.

Capillary HemoCue measurements are known to be imperfect[2,3]. Bell[2] et al compared haemoglobin measured using capillary HemoCue versus gold standard in 5724 women who were aged 18+ and eligible to give blood via the NHS donation service. We use Bell's analyses to correct for capillary HemoCue inaccuracy in our study.

Problem
For our study we use a logistic regression model [1] to calculate the odds ratio for the association between prebirth haemoglobin and postpartum haemorrhage (PPH).

$$logit\ (p_i) = \beta_0 + \beta_1 h_i \qquad \text{Equation 1}$$

p_i is the probability of PPH
h_i= prebirth haemoglobin measured with a HemoCue 201+

We require estimates of β_0 and β_1 corrected for HemoCue mismeasurement. If g_i is the gold standard haemoglobin measurement for patient i, we need estimates of β_0^* and β_1^* such that:

$$logit\ (p_i) = \beta_0^* + \beta_1^* g_i \qquad \text{Equation 2}$$

Our solution
Bell et al used Bland-Altman analyses to compare haemoglobin measured by capillary HemoCue versus gold standard.

Equation 3 describes the relationship modelled by Bell. The authors of Bell et al provided us with estimates of the means and the variance covariance matrix for α_0 and α_1 as well as the variance of the error term σ^2 (see below).

$$h_i - g_i = \alpha_0 + \alpha_1\left(\frac{g_i+h_i}{2}\right) + \epsilon_i \qquad \text{Equation 3}$$

where $\epsilon_i \sim N(0, \sigma^2)$

However, to correct for HemoCue mismeasurement we need the relationship between gold standard and capillary HemoCue as described by equation 4:

$$g_i = \alpha_0^* + \alpha_1^* h_i + \epsilon_i^* \qquad \text{Equation 4}$$

Rearranging equation 3 we can obtain estimates for α_0^*, α_0^* and ϵ_i^*.

$$\alpha_o^* = -\frac{\alpha_0}{1+\frac{\alpha_1}{2}}$$
$$\alpha_1^* = \frac{1-\frac{\alpha_1}{2}}{1+\frac{\alpha_1}{2}}$$

$$\epsilon_i^* = -\frac{\epsilon_i}{1+\frac{\alpha_1}{2}}$$

To obtain estimates for β_0^* and β_1^* we repeat the following steps 1000 times:

1. Simulate random errors ϵ_i, i=1,….,n where n is the sample size.
2. Simulate α_0 and α_1 using a bivariate normal distribution

3. Calculate the corresponding values of α_0^*, α_1^* and ϵ_i^*
4. Calculate estimates of the gold standard measurement using equation (4)
5. To accommodate uncertainty in the estimates of β_0 and β_1 we randomly sample with replacement ("bootstrap") our dataset.
6. We obtain maximum likelihood estimates for β_0^* and β_1^* using equation 2 and our bootstrap sample dataset.

Information received by private communication

The authors of Bell et al provided us with the following parameters via email on 21st January 2022

$\alpha_0 = -18.86\ g/L$
$\alpha_1 = 0.127$
$\sigma = 7.27$ g/L

Variance covariance matrix from equation (3)

	α_0	α_1
α_0	1.436	-0.0110
α_1	-0.0110	0.0000842

Web appendix table 1a: Baseline characteristics of women with moderate or severe anaemia from sites in Pakistan. WOMAN-2 trial. N=8751. For hypertensive disease, infection, assisted delivery, augmentation and induction proportions do not add to 100% as responses are not mutually exclusive. For other variables proportions may not sum to 100% due to rounding.

Variable	Number	% (SD)
**Haemoglobin (g/L)**		
Mean (SD)	79.6	(11.7)
Moderately anaemic	7113	81.3 %
Severely anaemic	1638	18.7 %
**Age (Years)**		
Mean (SD)	27.1	(5.4)
<20	407	4.7 %
20-29	5337	61.0 %
30-39	2788	31.9 %
40+	219	2.5 %
**BMI (kg/m^2)**		
Mean (SD)	26.4	(3.7)
<25	3260	37.3 %
25-30	4128	47.2 %
30-39	1346	15.4 %
40+	16	0.2 %
Missing	1	< 0.1 %
**Weight (kg)**		
Mean (SD)	66	(8.8)
<55	682	7.8 %
55-64	3025	34.6 %
65-74	3570	40.8 %
≥75	1474	16.8 %
**Number of foetuses**		
1	8486	97.0 %
2	255	2.9 %
3	10	0.1 %
**Parity (includes this pregnancy)**		
1	2781	31.8 %
2-4	4101	46.9 %
5+	1869	21.4 %
**Previous PPH**		
Yes	77	0.9 %
No	5754	65.8 %
No previous birth	2779	31.8 %
Missing	141	1.6 %

Variable	Number	% (SD)
**Placenta abnormalities**		
Abruption	302	3.5 %
Previa	27	0.3 %
Abruption, previa	5	0.1 %
Accreta	0	0.0 %
None	8417	96.2 %
**Any antepartum haemorrhage**		
Yes	300	3.4 %
No	8451	96.6 %
**Number of previous c-sections**		
0	8285	94.7 %
1	441	5.0 %
2	19	0.2 %
3	6	0.1 %
**Current infection status**		
HIV	2	< 0.1 %
Hepatitis	79	0.9 %
Malaria	1	< 0.1 %
Syphilis	0	0.0 %
Other	51	0.6 %
None	8620	98.5 %
**Hypertensive disease**		
Eclampsia	23	0.3 %
Pre-eclampsia	144	1.6 %
Pre-existing hypertension	39	0.4 %
Pregnancy-induced hypertension	465	5.3 %
None	8083	92.4 %
**Assisted delivery**		
Forceps	88	1.0 %
Ventouse	120	1.4 %
Other	32	0.4 %
None	8515	97.3 %
**Induction**		
Artificial membrane rupture	279	3.2 %
Mechanical method	357	4.1 %
Membrane sweep	151	1.7 %
Oxytocin	43	0.5 %
Misoprostol	227	2.6 %
Prostaglandin	376	4.3 %
None	7542	86.2 %

Variable	Number	% (SD)
**Augmentation**		
Oxytocin	2515	28.7 %
Other	244	2.8 %
None	6045	69.1 %
**Episiotomy**		
Yes	2997	34.2 %
No	5754	65.8 %
**Long labour**		
Yes	478	5.5 %
No	8273	94.5 %
**Known macrosomia**		
Yes	114	1.3 %
No	8637	98.7 %

Web appendix table 1b: Baseline characteristics of women with moderate or severe anaemia from sites in Nigeria. WOMAN-2 trial. N=837. For hypertensive disease, infection, assisted delivery, augmentation and induction proportions do not add to 100% as responses are not mutually exclusive. For other variables proportions may not sum to 100% due to rounding.

Variable	Number	% (SD)
**Haemoglobin (g/L)**		
Mean (SD)	85.9	(8.4)
Moderately anaemic	791	94.5 %
Severely anaemic	46	5.5 %
**Age (Years)**		
Mean (SD)	27.8	(5.8)
<20	49	5.9 %
20-29	473	56.5 %
30-39	289	34.5 %
40+	26	3.1 %
**BMI (kg/m^2)**		
Mean (SD)	26.7	(4.0)
<25	303	36.2 %
25-30	395	47.2 %
30-39	132	15.8 %
40+	6	0.7 %
Missing	1	0.1 %
**Weight (kg)**		
Mean (SD)	68.6	(10.9)
<55	71	8.5 %
55-64	210	25.1 %
65-74	339	40.5 %
≥75	217	25.9 %
**Number of foetuses**		
1	800	95.6 %
2	37	4.4 %
**Parity (includes this pregnancy)**		
1	334	39.9 %
2-4	417	49.8 %
5+	86	10.3 %
**Previous PPH**		
Yes	20	2.4 %
No	481	57.5 %
No previous birth	332	39.7 %
Missing	4	0.5 %

Variable	Number	% (SD)
**Placenta abnormalities**		
Abruption	7	0.8 %
Previa	2	0.2 %
Abruption, previa	0	0.0 %
Accreta	1	0.1 %
None	827	98.9 %
**Any antepartum haemorrhage**		
Yes	12	1.4 %
No	825	98.6 %
**Number of previous c-sections**		
0	807	96.4 %
1	29	3.5 %
2	1	0.1 %
**Current infection status**		
HIV	10	1.2 %
Hepatitis	36	4.3 %
Malaria	12	1.4 %
Syphilis	2	0.2 %
Other	2	0.2 %
None	777	92.8 %
**Hypertensive disease**		
Eclampsia	3	0.4 %
Pre-eclampsia	8	1.0 %
Pre-existing hypertension	4	0.5 %
Pregnancy-induced hypertension	32	3.8 %
None	790	94.4 %
**Assisted delivery**		
Forceps	4	0.5 %
Ventouse	4	0.5 %
Other	3	0.4 %
None	826	98.7 %
**Induction**		
Artificial membrane rupture	31	3.7 %
Mechanical method	15	1.8 %
Membrane sweep	2	0.2 %
Oxytocin	1	0.1 %
Misoprostol	66	7.9 %
Prostaglandin	0	0.0 %
None	734	87.7 %
**Augmentation**		
Oxytocin	343	41.0 %
Other	7	0.8 %
None	491	58.7 %

Variable	Number	% (SD)
**Episiotomy**		
Yes	248	29.6 %
No	589	70.4 %
**Long labour**		
Yes	140	16.7 %
No	697	83.3 %
**Known macrosomia**		
Yes	28	3.3 %
No	809	96.7 %

Web appendix table 1c: Baseline characteristics of women with moderate or severe anaemia from sites in Tanzania. WOMAN-2 trial. N=525. For hypertensive disease, infection, assisted delivery, augmentation and induction proportions do not add to 100% as responses are not mutually exclusive. For other variables proportions may not sum to 100% due to rounding.

Variable	Number	% (SD)
**Haemoglobin (g/L)**		
Mean (SD)	87.3	(11.2)
Moderately anaemic	485	92.4 %
Severely anaemic	40	7.6 %
**Age (Years)**		
Mean (SD)	26.3	(6.0)
<20	59	11.2 %
20-29	314	59.8 %
30-39	141	26.9 %
40+	11	2.1 %
**BMI (kg/m^2)**		
Mean (SD)	26.7	(4.6)
<25	211	40.2 %
25-30	219	41.7 %
30-39	85	16.2 %
40+	10	1.9 %
**Weight (kg)**		
Mean (SD)	67.3	(11.4)
<55	49	9.3 %
55-64	182	34.7 %
65-74	183	34.9 %
≥75	111	21.1 %
**Number of foetuses**		
1	492	93.7 %
2	33	6.3 %
**Parity (includes this pregnancy)**		
1	217	41.3 %
2-4	255	48.6 %
5+	53	10.1 %
**Previous PPH**		
Yes	8	1.5 %
No	294	56.0 %
No previous birth	217	41.3 %
Missing	6	1.1 %

Variable	Number	% (SD)
**Placenta abnormalities**		
Abruption	8	1.5 %
Previa	0	0.0 %
Abruption, previa	0	0.0 %
Accreta	0	0.0 %
None	517	98.5 %
**Any antepartum haemorrhage**		
Yes	7	1.3 %
No	518	98.7 %
**Number of previous c-sections**		
0	510	97.1 %
1	15	2.9 %
**Current infection status**		
HIV	30	5.7 %
Hepatitis	1	0.2 %
Malaria	1	0.2 %
Syphilis	0	0.0 %
Other	3	0.6 %
None	491	93.5 %
**Hypertensive disease**		
Eclampsia	1	0.2 %
Pre-eclampsia	23	4.4 %
Pre-existing hypertension	0	0.0 %
Pregnancy-induced hypertension	14	2.7 %
None	487	92.8 %
**Assisted delivery**		
Forceps	2	0.4 %
Ventouse	34	6.5 %
Other	1	0.2 %
None	488	93.0 %
**Induction**		
Artificial membrane rupture	7	1.3 %
Mechanical method	1	0.2 %
Membrane sweep	1	0.2 %
Oxytocin	1	0.2 %
Misoprostol	5	1.0 %
Prostaglandin	10	1.9 %
None	502	95.6 %
**Augmentation**		
Oxytocin	193	36.8 %
Other	3	0.6 %
None	330	62.9 %

Variable	Number	% (SD)
**Episiotomy**		
Yes	61	11.6 %
No	464	88.4 %
**Long labour**		
Yes	76	14.5 %
No	449	85.5 %
**Known macrosomia**		
Yes	15	2.9 %
No	510	97.1 %

Web appendix table 1d: Baseline characteristics of women with moderate or severe anaemia from sites in Zambia. WOMAN-2 trial. N= 448. For hypertensive disease, infection, assisted delivery, augmentation and induction proportions will not add to 100% as responses are not mutually exclusive. For other variables proportions may not sum to 100% due to rounding.

Variable	Number	% (SD)
**Haemoglobin (g/L)**		
Mean (SD)	85.4	(12.4)
Moderately anaemic	402	89.7 %
Severely anaemic	46	10.3 %
**Age (Years)**		
Mean (SD)	27	(6.8)
<20	57	12.7 %
20-29	237	52.9 %
30-39	137	30.6 %
40+	17	3.8 %
**BMI (kg/m^2)**		
Mean (SD)	27	(4.6)
<25	159	35.5 %
25-30	182	40.6 %
30-39	102	22.8 %
40+	5	1.1 %
**Weight (kg)**		
Mean (SD)	65	(12.1)
<55	73	16.3 %
55-64	165	36.8 %
65-74	116	25.9 %
≥75	94	21.0 %
**Number of foetuses**		
1	409	91.3 %
2	38	8.5 %
3	1	0.2 %
**Parity (includes this pregnancy)**		
1	158	35.3 %
2-4	211	47.1 %
5+	79	17.6 %
**Previous PPH**		
Yes	8	1.8 %
No	279	62.3 %
No previous birth	158	35.3 %
Missing	3	0.7 %

Variable	Number	% (SD)
**Placenta abnormalities**		
Abruption	15	3.3 %
Previa	2	0.4 %
Abruption, previa	0	0.0 %
Accreta	0	0.0 %
None	431	96.2 %
**Any antepartum haemorrhage**		
Yes	18	4.0 %
No	430	96.0 %
**Number of previous c-sections**		
0	421	94.0 %
1	27	6.0 %
**Current infection status**		
HIV	131	29.2 %
Hepatitis	2	0.4 %
Malaria	2	0.4 %
Syphilis	9	2.0 %
Other	16	3.6 %
None	297	66.3 %
**Hypertensive disease**		
Eclampsia	0	<0.1 %
Pre-eclampsia	29	6.5 %
Pre-existing hypertension	2	0.4 %
Pregnancy-induced hypertension	29	6.5 %
None	389	86.8 %
**Assisted delivery**		
Forceps	2	0.4 %
Ventouse	2	0.4 %
Other	17	3.8 %
None	427	95.3 %
**Induction**		
Artificial membrane rupture	11	2.5 %
Mechanical method	7	1.6 %
Membrane sweep	1	0.2 %
Oxytocin	0	0.0 %
Misoprostol	43	9.6 %
Prostaglandin	0	<0.1 %
None	389	86.8 %
**Augmentation**		
Oxytocin	111	24.8 %
Other	5	1.1 %
None	335	74.8 %
**Episiotomy**		
Yes	64	14.3 %
No	384	85.7 %

Variable	Number	% (SD)
**Long labour**		
Yes	33	7.4 %
No	415	92.6 %
**Known macrosomia**		
Yes	5	1.1 %
No	443	98.9 %

Appendix table 2: Univariable and multivariable analyses of risk factors for clinical postpartum haemorrhage in women with moderate or severe anaemia. The multivariable model controls for haemoglobin, country, BMI, number of foetuses, parity, previous PPH, previous caesarean sections, hypertensive disease, assisted delivery, augmentation/induction, episiotomy, long labour and known macrosomia. N=10561 for the univariable model and N=10405 for the multivariable model. Abbreviations: aOR (adjusted odds ratio)

Variable	Total	Number of women with PPH (%)	OR (95% CI)	aOR (95% CI)*
**Haemoglobin (10 g/L)**	10561	742 (7.0)	1.36 (1.27-1.46)	1.40 (1.30-1.50)
**Country**				
Pakistan	8751	601 (6.9)	1.00	1.00
Nigeria	837	78 (9.3)	1.47 (0.99-2.19)	2.01 (1.30-3.12)
Tanzania	525	32 (6.1)	0.95 (0.62-1.46)	1.34 (0.85-2.13)
Zambia	448	31 (6.9)	1.11 (0.90-1.38)	1.37 (1.06-1.76)
**Body mass index kg/m²**				
<25	3933	284 (7.2)	1.00	1.00
25-30	4924	337 (6.8)	0.99 (0.84-1.18)	0.99 (0.81-1.21)
30-39	1665	117 (7.0)	1.04 (0.78-1.38)	0.95 (0.69-1.30)
40+	37	4 (10.8)	1.60 (0.51-5.04)	1.37 (0.37-5.11)
**Number of foetuses**				
1	10187	672 (6.6)	1.00	1.00
2+	374	70 (18.7)	3.36 (2.58-4.38)	3.46 (2.62-4.58)
**Parity (includes this pregnancy)**				
1	3490	251 (7.2)	1.00	1.00
2-4	4984	310 (6.2)	0.84 (0.71-1.00)	1.05 (0.86-1.29)
5+	2087	181 (8.7)	1.15 (0.92-1.44)	1.36 (1.06-1.75)
**Previous postpartum haemorrhage**				
No or no previous birth	10294	707 (6.9)	1.00	1.00
Yes	113	17 (15.0)	2.34 (1.27-4.32)	2.23 (1.26-3.94)
**Previous c-section(s)**				
No	10023	708 (7.1)	1.00	1.00
Yes	538	34 (6.3)	0.88 (0.58-1.33)	0.83 (0.56-1.23)
**Hypertensive disease**				
No	9749	610 (6.3)	1.00	1.00
Yes	812	132 (16.3)	3.01 (2.32-3.89)	2.77 (2.12-3.63)
**Assisted delivery**				
No	10256	688 (6.7)	1.00	1.00
Yes	305	54 (17.7)	3.10 (2.25-4.27)	2.28 (1.59-3.29)
**Augmentation/Induction**				
No	6612	413 (6.2)	1.00	1.00
Yes	3949	329 (8.3)	1.41 (1.15-1.74)	1.41 (1.11-1.79)
**Episiotomy**				
No	7191	480 (6.7)	1.00	1.00
Yes	3370	262 (7.8)	1.25 (1.03-1.51)	1.49 (1.16-1.90)

Variable	Total	Number of women with PPH (%)	OR (95% CI)	aOR (95% CI)*
**Long labour**				
No	9834	682 (6.9)	1.00	1.00
Yes	727	60 (8.3)	1.13 (0.91-1.42)	1.08 (0.85-1.38)
**Known macrosomia**				
No	10399	719 (6.9)	1.00	1.00
Yes	162	23 (14.2)	2.21 (1.47-3.32)	2.24 (1.52-3.31)

References

1 Say L, Souza JP, Pattinson RC. Maternal near miss – towards a standard tool for monitoring quality of maternal health care. *Best Practice & Research Clinical Obstetrics & Gynaecology* 2009; **23**: 287–96.

2 Bell S, Sweeting M, Ramond A, *et al.* Comparison of four methods to measure haemoglobin concentrations in whole blood donors (COMPARE): A diagnostic accuracy study. *Transfusion Medicine* 2021; **31**: 94–103.

3 Patel AJ, Wesley R, Leitman SF, Bryant BJ. Capillary versus venous haemoglobin determination in the assessment of healthy blood donors. *Vox Sanguinis* 2013; **104**: 317–23.
